# Shared and unique effects of *ApoEε4* and pathogenic gene mutation on cognition and imaging in preclinical familial Alzheimer’s disease

**DOI:** 10.1186/s13195-023-01192-y

**Published:** 2023-02-28

**Authors:** Meina Quan, Qi Wang, Wei Qin, Wei Wang, Fangyu Li, Tan Zhao, Tingting Li, Qiongqiong Qiu, Shuman Cao, Shiyuan Wang, Yan Wang, Hongmei Jin, Aihong Zhou, Jiliang Fang, Longfei Jia, Jianping Jia

**Affiliations:** 1 Innovation Center for Neurological Disorders and Department of Neurology, Xuanwu Hospital, Capital Medical University, Beijing, China; 2 National Center for Neurological Disorders and National Clinical Research Center for Geriatric Diseases, Beijing, China; 3 Clinical Center for Neurodegenerative Disease and Memory Impairment, Capital Medical University, Beijing, China; 4 Beijing Key Laboratory of Geriatric Cognitive Disorders, Beijing, China; 5 Center of Alzheimer’s Disease, Beijing Institute for Brain Disorders, Beijing, China; 6 Key Laboratory of Neurodegenerative Diseases, Ministry of Education, Beijing, China; 7 Guang’anmen Hospital, China Academy of Chinese Medical Sciences, Beijing, China

**Keywords:** Familial Alzheimer’s disease, Gene mutation, Neuropsychology, Diffusion tensor imaging, Resting state functional MRI

## Abstract

**Background:**

Neuropsychology and imaging changes have been reported in the preclinical stage of familial Alzheimer’s disease (FAD). This study investigated the effects of *APOEε4* and known pathogenic gene mutation on different cognitive domains and circuit imaging markers in preclinical FAD.

**Methods:**

One hundred thirty-nine asymptomatic subjects in FAD families, including 26 *APOEε4* carriers, 17 *APP* and 20 *PS1* mutation carriers, and 76 control subjects, went through a series of neuropsychological tests and MRI scanning. Test scores and imaging measures including volumes, diffusion indices, and functional connectivity (FC) of frontostriatal and hippocampus to posterior cingulate cortex pathways were compared between groups and analyzed for correlation.

**Results:**

Compared with controls, the *APOEε4* group showed increased hippocampal volume and decreased FC of fronto-caudate pathway. The *APP* group showed increased recall scores in auditory verbal learning test, decreased fiber number, and increased radial diffusivity and FC of frontostriatal pathway. All three genetic groups showed decreased fractional anisotropy of hippocampus to posterior cingulate cortex pathway. These neuropsychological and imaging measures were able to discriminate genetic groups from controls, with areas under the curve from 0.733 to 0.837. Circuit imaging measures are differentially associated with scores in various cognitive scales in control and genetic groups.

**Conclusions:**

There are neuropsychological and imaging changes in the preclinical stage of FAD, some of which are shared by *APOEε4* and known pathogenic gene mutation, while some are unique to different genetic groups. These findings are helpful for the early identification of Alzheimer’s disease and for developing generalized and individualized prevention and intervention strategies.

**Supplementary Information:**

The online version contains supplementary material available at 10.1186/s13195-023-01192-y.

## Background

Alzheimer’s disease, the major type of dementia, is a serious challenge for aging society worldwide, including in China [1]. Familial Alzheimer’s disease (FAD) accounts for 15–25% of total Alzheimer’s disease and has presented a useful model for studying the pathogenesis and trajectory of the disorder [2, 3]. Those carrying known causative gene mutations including the amyloid precursor protein (*APP*), presenilin 1 (*PS1*), or presenilin 2 (*PS2*) are nearly 100% certain to show sequential clinical features and biomarker changes and thus can be diagnosed before symptoms onset. Besides known causative gene mutation, the ε4 allele of the apolipoprotein E gene (*APOE*) is the strongest genetic risk factor for sporadic Alzheimer’s disease [4]. Interestingly, a recent study found that the genetic risk effect of *APOE*ε4 is higher in FAD with unknown mutation than in sporadic Alzheimer’s disease [5]. Thus, further study of the *APOE*ε4 effect in FAD in comparison with those known pathogenic gene mutations would be helpful in understanding the commonality and heterogeneity in pathogenesis.

Studies have shown neuropsychological and MRI imaging changes in the preclinical stage of FAD. Several cognitive domains are impaired in the preclinical stage, namely episodic memory, executive function, and long-term forgetting [6, 7]. Studies also confirmed the early volumetric changes of the striatum [8, 9] and hippocampus [10, 11], finer structural changes of the striatum and hippocampus relative to controls in diffusion tensor imaging (DTI) [8], and early changes of striatum or hippocampus activity in functional MRI [12, 13]. Interestingly, striatum and hippocampus related neural circuits are involved in many aforementioned cognitive domains. Specifically, the frontostriatal circuit plays a critical role in executive function and working memory [14, 15]. Hippocampus-PCC circuit and medial-temporal atrophy including the hippocampus are related to episodic memory and language domain in the pre-dementia stage [16, 17].

Although numerous neuropsychological and imaging findings suggest the early changes of different cognitive domains and striatum and hippocampus-related imaging markers in the preclinical stage of FAD, most of the findings have involved mutation carriers of various genes and have not looked at the effect of specific gene or *APOE*ε4. Limited evidence suggests the pathogenic gene-specific effect [18] or *APOE*ε4-specific effect [19] on different cognitive domains as compared with non-carriers in familial or mild sporadic Alzheimer’s disease. Also, limited evidence shows the pathogenic gene-specific effect [20, 21] or *APOE*ε4-specific effect [22] on MRI imaging in Alzheimer’s disease. However, seldom has shown the specific effect in preclinical stages of Alzheimer’s disease.

This study was aimed at exploring the effects of *APOE*ε4 and known pathogenic gene mutations (*PS1* and *APP*) on different cognitive domains and the structural and functional connectivity of frontostriatal and hippocampus-PCC circuits in preclinical FAD. The results in their commonality and heterogeneity may shed light on early identification of Alzheimer’s disease and pave the way for patient selection in clinical trials as well as the development of population-based or individualized intervention or prevention strategies.

## Methods

### Participants

All the participants were recruited from two ongoing cohort studies called the Chinese Familial Alzheimer’s Disease Network study (CFAN, Study ID Number: SYXWJ002; ClinicalTrials.gov Identifier: NCT03657732) and China Cognition and Aging Study (COAST, Study ID Number: SYXWJ001; ClinicalTrials.gov Identifier: NCT03653156) that receive research referrals from across China. FAD was defined as at least one first-degree relative in addition to the patient himself/herself within the family who had objective cognitive decline suggestive of Alzheimer’s disease [2]. All procedures contributing to this work comply with the ethical standards at Xuanwu Hospital on human experimentation and with the Helsinki Declaration of 1975, as revised in 2013.

The inclusion criteria are as follows: all subjects had undergone clinical diagnosis and were aware of their mutation status. All subjects in the study underwent general cognitive assessments including the Mini-Mental State Examination (MMSE) [23] and the Montreal Cognitive Assessment (MoCA) [24] for general cognitive functions and the Clinical Dementia Rating scale (CDR) [25] for clinical symptoms. The CDR global score was required to be zero. Estimated years from symptom onset (EYO) was calculated by subtracting the mean family age at symptoms onset from his/her current age [10].

The exclusion criteria are as follows: participants who exhibited any condition that might preclude completion of neuropsychological testing or MRI scanning were excluded. Those with infarcts, hemorrhages, stroke, vascular disease, hydrocephalus, white matter lesions, or hyperintensities were excluded. Those with psychiatric conditions namely psychosis, depression, and anxiety were excluded using neuropsychiatric assessments for psychiatric symptoms, including neuropsychiatric inventory (NPI-Q) [26], Hamilton anxiety rating scale (HAMA) [27, 28], and Hamilton depression scale (HAMD) [29, 30].

**Fig. 1:**
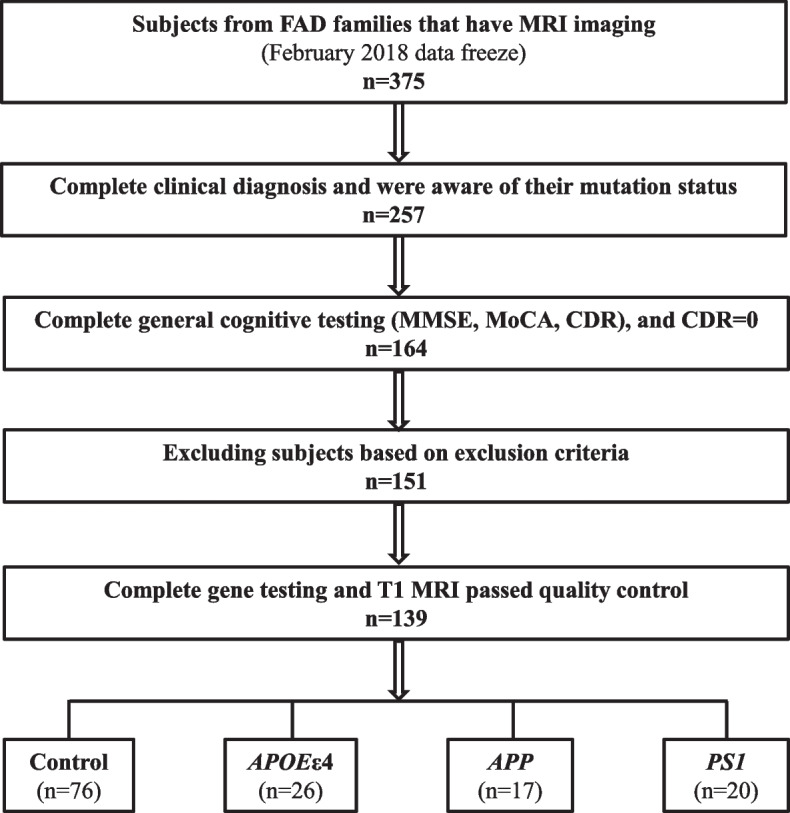
The diagram of subject selection and exclusion procedures

We included 37 asymptomatic subjects carrying known gene mutations and 102 cognitive normal subjects within the FAD pedigrees who do not carry the known pathogenic gene mutations. Among them, 17 carried *APP* mutation, 20 carried *PS1* mutation, 26 carried *APOE*ε4, and 76 controls did not carry *APOE*ε4 (Fig. 1).

### Gene testing

After informed consent of the study subjects, 3 ml of the peripheral venous blood of the participants were drawn by venipuncture. Peripheral blood genomic DNA was extracted by salting-out procedures as previously described [31]. *APOE*, *PS1*, *PS2*, and *APP* gene primers were designed as previously described [5]. *APOE*, *PS1*, *PS2*, and *APP* gene are screened by PCR. The PCR products were subjected to sequencing using an ABI3730xl DNA Analyzer (Sangon Biotech Co., Ltd., Shanghai, China). The DNA sequencing results were analyzed using Chromas (Chromas version 2.33, Technelysium Pty Ltd, USA). The pathogenicity of the detected mutations in *PS1*, *PS2*, or *APP* was assessed using the Alzheimer’s disease Mutation Database (http://www.molgen.ua.ac.be/ADMutations/), AlzForum (http://www.alzforum.org/), PubMed (http://www.ncbi.nlm.nih.gov/), PolyPhen-2 (http://genetics.bwh.harvard.edu/pph2/), and Mutation Taster (http://www.mutationtaster.org).

### Neuropsychological assessment

All subjects underwent neuropsychological assessments for different cognitive domains, including auditory verbal learning Test (AVLT), which consists of immediate, cued, and delayed recall and recognition, that reflect verbal working and episode memory [32]; Rey-Osterrieth complex figure test (ROCF), which consists of a copy trial and a recall trial of a complex figure, that reflect visuospatial working and episode memory [33]; digit span test-forward and backward, which tests number storage capacity in working memory [34]; trail making test (TMT) A and B, which reflects executive function, perceptual scanning skills and cognitive flexibility [35]; and Boston naming test (BNT), which reflects language/semantic memory [36].

### Image acquisition

All subjects were scanned on the same 3.0 T Siemens Skyra scanner (Germany) using a 20-channel phased array head-neck coil. Whole-brain T1-weighted three-dimensional (3D) magnetization-prepared rapid gradient echo (MPRAGE) scans were acquired. Whole-brain 30-direction spin-echo echo planar imaging (EPI) sequence of DTI scans with an anterior-to-posterior phase-encoding direction were acquired. Twelve reference volume (*b* = 0 s/mm^2^) and 90 diffusion volumes (*b* = 1000 s/mm^2^) with uniformly distributed diffusion directions were acquired. Resting-State fMRI (RsfMRI) scans were collected using a gradient EPI sequence. Participants were required to keep their eyes open during the resting-state scanning. The scanning parameters for T1, DTI, and rsfMRI were the same as in our previously published study [37]. Imaging data were stored in DICOM format (.dcm), and converted to nifty format (.nii) using dcm2nii software for processing. Subjects had T1, DTI, and rsfMRI imaging scans from the same scanner within one scanning period, and the T1 image passed quality control. Further quality control was done for DTI and rsfMRI scans. Those DTI scans with incomplete coverage of the brain due to a restricted field of view or significant motion affecting the intensity were excluded. Those rsfMRI scans with incomplete coverage of the brain due to a restricted field of view, motion affected the intensity, or missing a certain number of volumes were excluded.

### Image processing

T1 images were preprocessed using fslmaths and FreeSurfer software. DTI images were preprocessed using the FSL software. RsfMRI images were preprocessed using the SPM and BRANT software. Details of preprocessing could be found in our previously published study [37]. Briefly, T1 images for each subject were preprocessed using fslmaths command with a threshold of 80 for background noise reduction and using the FreeSurfer software package version 5.3.0 for brain extraction and segmentation [38]. DTI images were preprocessed using the FSL software FDT toolbox, including BET for brain mask generation, Eddy correct for correction of eddy current distortions, DTIFIT for head motion correction and reconstruction of diffusion tensors, and Bedpostx for local modeling of diffusion parameters [39]. Then, brain-extracted DTI images were registered with betted and non-betted T1 image of the same subject and Montreal Neurological Institute (MNI) standard space image using Registration module. The regions of interest (ROIs) were reoriented from FreeSurfer space to structural space and registered to the diffusion space using FLIRT, with nearest neighbor interpolation, and to the MNI standard space using FNIRT. The data of rsfMRI were preprocessed by SPM12 [40] and BRANT [41], following steps including slice timing correction, head motion correction, co-registration of segmented T1 image with the mean rsfMRI image, spatial normalization, spatial smoothing using Gaussian kernel with full-width at half maximum of 6 mm, regressing out linear trend, mean time series extracted from tissue masks and six head motion parameters, and temporal filtering using a 0.01–0.08 Hz band-pass filter.

After preprocessing, ten ROIs were obtained from T1 data for each subject, including bilateral caudate, putamen, hippocampus, PCC, and rostral middle frontal gyrus (rMFG), which likely represents DLPFC [42]. The volume (absolute volume) of each ROI was calculated from the FreeSurfer software automatically. Then, the relative volume was calculated as the percentage of absolute volume in intracranial volume, to correct the effect of difference in brain size among subjects. The DTI data were analyzed using the Probtrack (probabilistic tracking) module in the FSL software. Bilateral caudate, putamen, and hippocampus were set as seed ROIs separately, and bilateral PCC and rMFG were set as waypoints masks separately. At the end, six white matter tracts (fdt paths) were obtained, including bilateral caudate-rMFG, putamen-rMFG, and hippocampus-PCC tracts. Masks for each tract were generated with the threshold of 100. Diffusion parameters including fractional anisotropy (FA), mean diffusivity (MD), axial diffusivity (AxD), and radial diffusivity (RD) were measured using fslstats command. Fiber numbers were obtained from the waytotal output file. A seed-based approach was performed on rsfMRI data to calculate the functional connectivity (FC). Mean rsfMRI signals were extracted from each ROI separately by averaging the time courses signals of all voxels within the ROI. Pearson’s correlation coefficients (*r* values) were computed between caudate, putamen, and rMFG and between hippocampus and PCC and then transformed to *z* values to make it in accordance with Gaussian distribution. *Z* values of each pair of ROIs represent FC.

### Statistical analysis

All statistical analyses were performed using SPSS 22.0. For demographic data, continuous variables were compared among groups (control, *APOE*ε4, *APP*, *PS1*) using one-way ANOVA with post hoc between-group comparisons using Bonferroni analysis. Categorical variables were compared between groups (*APOE*ε4 vs. control, *APP* vs. control, *PS1* vs. control) using the chi-square test. The significant level was set at *P* < 0.05. For imaging data including relative volume of each ROI, diffusion parameters and FC of each tract, outliers (> mean + 2SD or < mean − 2SD) were excluded first from each group. Then, neuropsychological and imaging data were compared between groups (*APOE*ε4 vs. control, *APP* vs. control, *PS1* vs. control) using UNIANOVA, controlling for age, sex, and education, to see the effect of specific genetic markers on cognitive domains and imaging. Those neuropsychological and imaging measures that showed significant between-group differences were used to generate receiver operating characteristic (ROC) curves. The area under the curve (AUC) of ROC curves were analyzed to determine the ability of the neuropsychological and imaging measures to discriminate genetic groups from the control group. Bonferroni correction was used to correct for multiple comparisons. Significant level was set at *P* < 0.05/3 = 0.017. Trend level of significance was set at 0.017 < *P* < 0.1. Then, partial correlation analyses were performed for the imaging measures that showed group differences with neuropsychological measures, controlling for age, sex, and education. Bonferroni correction was used to correct for multiple comparisons. Significant level was set at *P* < 0.05/4 = 0.0125. Trend level of significance was set at 0.0125 < *P* < 0.1.

## Results

### Subject characteristics

Detailed demographic information for subjects is shown in Table 1. One-way ANOVA showed that there was a significant group difference in age (*P* = 0.003) and education (*P* = 0.034). Post hoc Bonferroni analysis showed that, as compared with the control group, the *PS1* group was younger (*P* = 0.014), while *APOEε4* and *APP* groups were similar in age (*P*’s > 0.05). As compared with the control group, the *APP* group was higher in education level (*P* = 0.037), while *APOEε4* and *PS1* groups were similar in education level (*P*’s > 0.05). The four groups were similar in sex and EYO (*P*’s > 0.05). In the *APOEε4* group, there was 1 subject with ε4/ε4, 22 subjects with ε4/ε3, and 3 subjects with ε4/ε2. Further analysis of the *APOEε4* subgroups showed no statistical difference of demographic information (Additional file 1: Table S1).

**Table 1:** Subject demographic and clinical data

	**Control**^a^ **(*n* = 76)**	***APOE*ε4** **(*n* = 26)**	***APP*** **(*n* = 17)**	***PS1*** **(*n* = 20)**	***P*-value**
Age (years)	44.37 (12.69)	44.54 (12.76)	35.65 (15.39)	33.95 (14.66)*	0.003**
Sex (male/female)	25/51	12/14	5/12	8/12	> 0.05
Education (years)	10.74 (5.48)	11.77 (4.68)	14.41 (2.27)*	10.25 (4.52)	0.034*
EYO (years)^b^	− 14.47 (11.08)	− 16.08 (9.77)	− 13.18 (13.73)	− 11.07 (13.38)	> 0.05
MMSE^c^	29.06 (0.18)	28.96 (0.30)	28.41 (0.37)	28.88 (0.35)	> 0.05
MoCA^d^	27.07 (2.26)	27.29 (2.53)	28.29 (1.57)	26.35 (3.56)	> 0.05
**Subject numbers**					
T1 MRI after PP^e^	70	24	17	20	
DTI after QC^f^ + PP	33	12	9	13	
rsfMRI after QC + PP	65	17	11	16	

For continuous variables, data are shown in mean (SD) and one-way ANOVA with post hoc between-group comparisons using Bonferroni analysis; for categorical variables, chi-square test was used to compare between groups (*APOEε4* vs. control, *APP* vs. control, *PS1* vs. control). *0.01 < *P* < 0.05, **0.001 < *P* < 0.01

^a^Control: cognitive normal subjects not carrying *APOEε4* or known pathogenic gene mutation

^b^EYO: estimated years from symptom onset

^c^MMSE = Mini-Mental State Examination

^d^MoCA = Montreal Cognitive Assessment

^e^PP: pre-processing

^f^QC: quality control

### Group comparisons of neuropsychological and imaging measures

Table 2 showed the group comparison results of neuropsychological measures. UNIANOVA showed that there were group differences for the AVLT. Specifically, compared with controls, *APP* subjects showed a significant increase in cued recall score (*P* = 0.008) and trend level increase in delayed recall score (*P* = 0.036). Other neuropsychological measures did not show group differences (*P*’s > 0.05). Since most subjects in the *APOEε4* group were ε4/ε3 (approximately 85%), and ε4/ε4 accounted for only 3.8%, further comparisons between *APOEε4* subgroups to explore the dose effect were not conducted. Further analysis using the 22 *APOEε4/ε3* subjects in the *APOEε4* group did not change the statistical difference as compared with the entire *APOEε4* group.

**Table 2:** Descriptive statistics of neuropsychological data

**Domain**	**Neuropsychological test**	**Subscale score**	**Control**^a^ **(*n* = 76)**	***APOE*ε4** **(*n* = 26)**	***APP*** **(*n* = 17)**	***PS1*** **(*n* = 20)**
**Episode memory**	AVLT^a^	Immediate 1	7.07 (2.68)	6.86 (2.17)	8.18 (2.13)	7.05 (1.43)
		Immediate 2	9.84 (2.67)	10.14 (2.53)	11.41 (1.84)	10.53 (1.62)
		Immediate 3	12.16 (2.25)	11.91 (2.33)	13.41 (1.73)	12.32 (1.80)
		Immediate total	28.93 (6.40)	28.86 (6.22)	33.12 (4.73)	29.89 (3.31)
		Delayed recall	10.87 (3.00)	11.59 (2.65)	**13.12 (1.58)#**	11.21 (2.32)
		Cued recall	10.49 (4.30)	10.50 (4.78)	13.59 (2.58)**	11.11 (3.68)
		Delayed recognition	12.73 (2.47)	13.05 (1.62)	14.06 (1.09)	12.42 (3.20)
	ROCF^b^	Figure copy	34.97 (3.11)	35.30 (1.61)	35.82 (0.53)	35.78 (0.55)
		Figure recall	21.14 (7.38)	21.14 (6.35)	21.12 (7.22)	21.03 (6.91)
**Working memory**	Digit Span	Forward	8.57 (1.21)	8.82 (1.22)	9.00 (0.61)	8.47 (1.07)
		Backward	5.85 (1.64)	6.05 (1.89)	6.24 (1.15)	5.79 (1.65)
**Language**	BNT^c^	Initial naming	26.25 (3.07)	27.36 (2.40)	27.12 (1.93)	25.58 (2.59)
		Cued naming	0.64 (0.98)	0.55 (0.96)	0.59 (1.50)	0.58 (0.77)
		Selective naming	2.73 (2.73)	1.77 (1.60)	2.35 (1.41)	3.32 (1.97)
**Visuospatial perception**	TMT^d^ A	Time (s)	37.66 (15.70)	36.23 (15.98)	33.18 (17.96)	30.36 (16.47)
		Correct trails	24.09 (1.26)	23.95 (0.21)	24.00 (0.00)	24.05 (0.23)
**Executive function**	TMT B	Time (s)	64.01 (38.89)	64.50 (37.51)	46.06 (17.05)	48.74 (26.90)
		Correct trails	23.55 (1.62)	23.09 (2.65)	24.00 (0.00)	24.05 (0.23)

^#^0.017 < *P* < 0.1, *0.01 < *P* < 0.017, **0.001 < *P* < 0.01

^a^AVLT: Auditory Verbal Learning Test

^b^ROCF: Rey-Osterrieth complex figure test

^c^BNT: Boston naming test

^d^TMT: trail making test

**Fig. 2:**
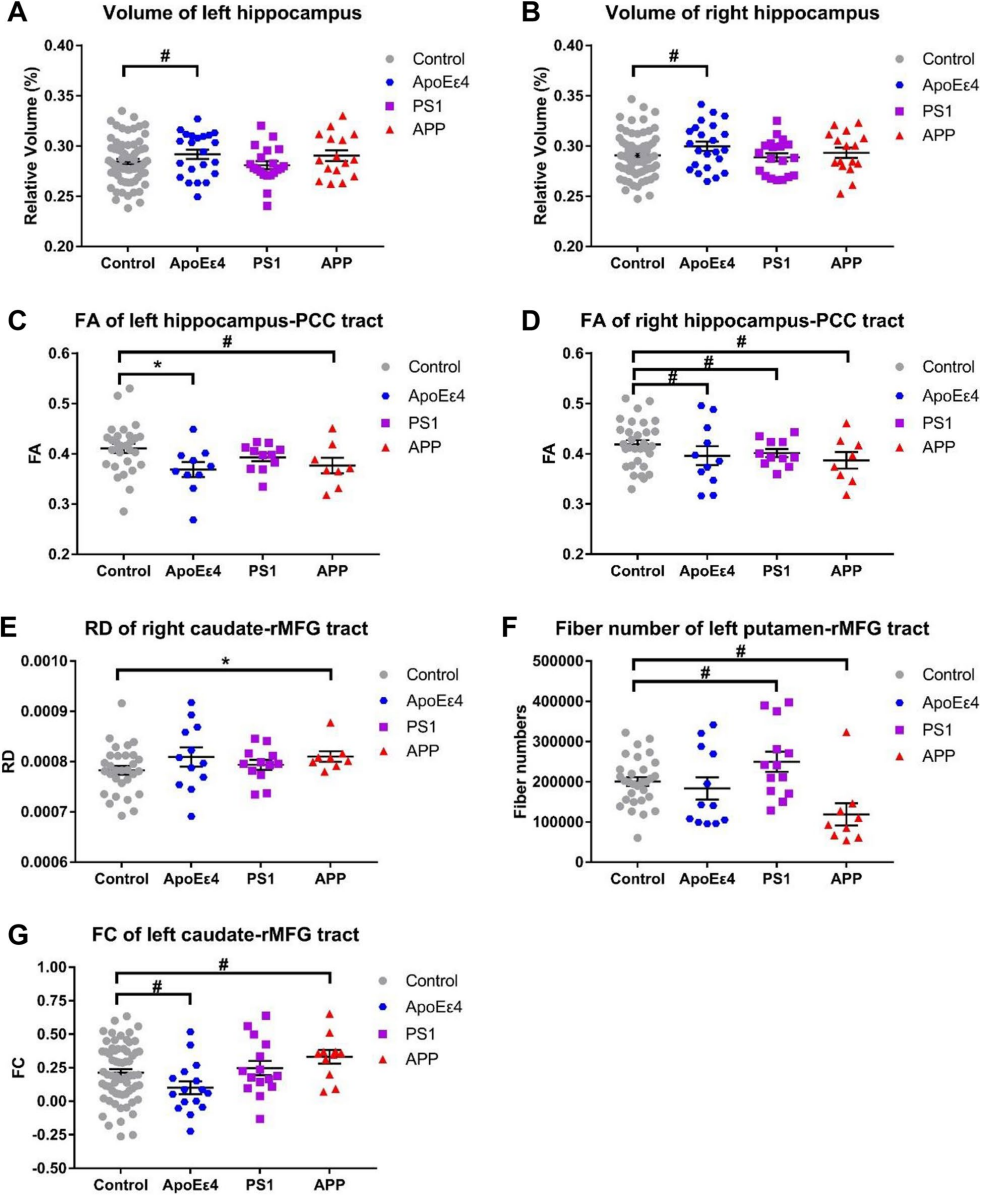
Group comparisons of the imaging measures. The bars indicate mean (SD). # 0.017 < *P* < 0.1, * 0.01 < *P* < 0.017

The representative ROIs and white matter tracts in diffusion and functional space can be found in our previous published paper [37]. The group comparison results of imaging measures were shown in Fig. 2. For ROI volumes, there was a trend level group difference in the relative volume of the hippocampus. Specifically, *APOEε4* group showed trend level increase in hippocampus (left: *P* = 0.065, right: *P* = 0.053) as compared with control group (Fig. 2A, B). For diffusion indices, there were group differences in FA of the hippocampus-PCC tract, the RD of the caudate-rMFG tract, and fiber number of putamen-rMFG tract. Specifically, for the FA of the left hippocampus-PCC tract, the *APOEε4* group showed a significant decrease (*P* = 0.015) and the *APP* group showed a trend level decrease (*P* = 0.086) as compared with controls (Fig. 2C); for the FA of right hippocampus-PCC tract, the *APOEε4* group (*P* = 0.074), the *APP* group (*P* = 0.037), and the *PS1* group (*P* = 0.059) showed trend level decrease as compared with the control group (Fig. 2D). For the RD of the right caudate-rMFG tract, the *APP* group showed a significant increase (*P* = 0.015) as compared with the control group (Fig. 2E). For the fiber number of the left putamen-rMFG tract, the *APP* group showed trend level decrease (*P* = 0.038), while the *PS1* group showed trend level increase (*P* = 0.051) as compared with the control group (Fig. 2F). For rsfMRI data, there were trend level group differences for the FC of the left caudate-rMFG tract. Specifically, the *APOEε4* group showed a trend level decrease (*P* = 0.059), while the *APP* group showed a trend level increase (*P* = 0.018) as compared with the control group (Fig. 2G).

### ROC curves of neuropsychological and neuroimaging measures

**Fig. 3:**
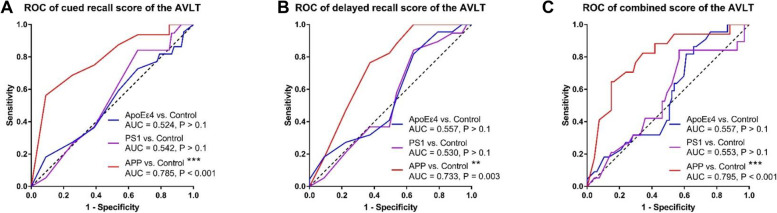
ROC curves of neuropsychological measures that showed group difference. # 0.017 < *P* < 0.1, * 0.01 < *P* < 0.017, ** 0.001 < *P* < 0.01, *** *P* < 0.001

The ROC curves of the neuropsychological measures that showed group differences were shown in Fig. 3. The AVLT cued recall score was able to discriminate the *APP* group from the control group (AUC = 0.785, *P* < 0.001) but not for the *APOEε4* or *PS1* groups (Fig. 3A). The AVLT delayed recall score was able to discriminate *APP* group from the control group (AUC = 0.733, *P* = 0.003), but not for the *APOEε4* or *PS1* groups (Fig. 3B). Combining cued recall score and delayed recall score in AVLT, it was able to discriminate the *APP* group from the control group (AUC = 0.795, *P* < 0.001), but not for the *APOEε4* or *PS1* groups (Fig. 3C).

**Fig. 4:**
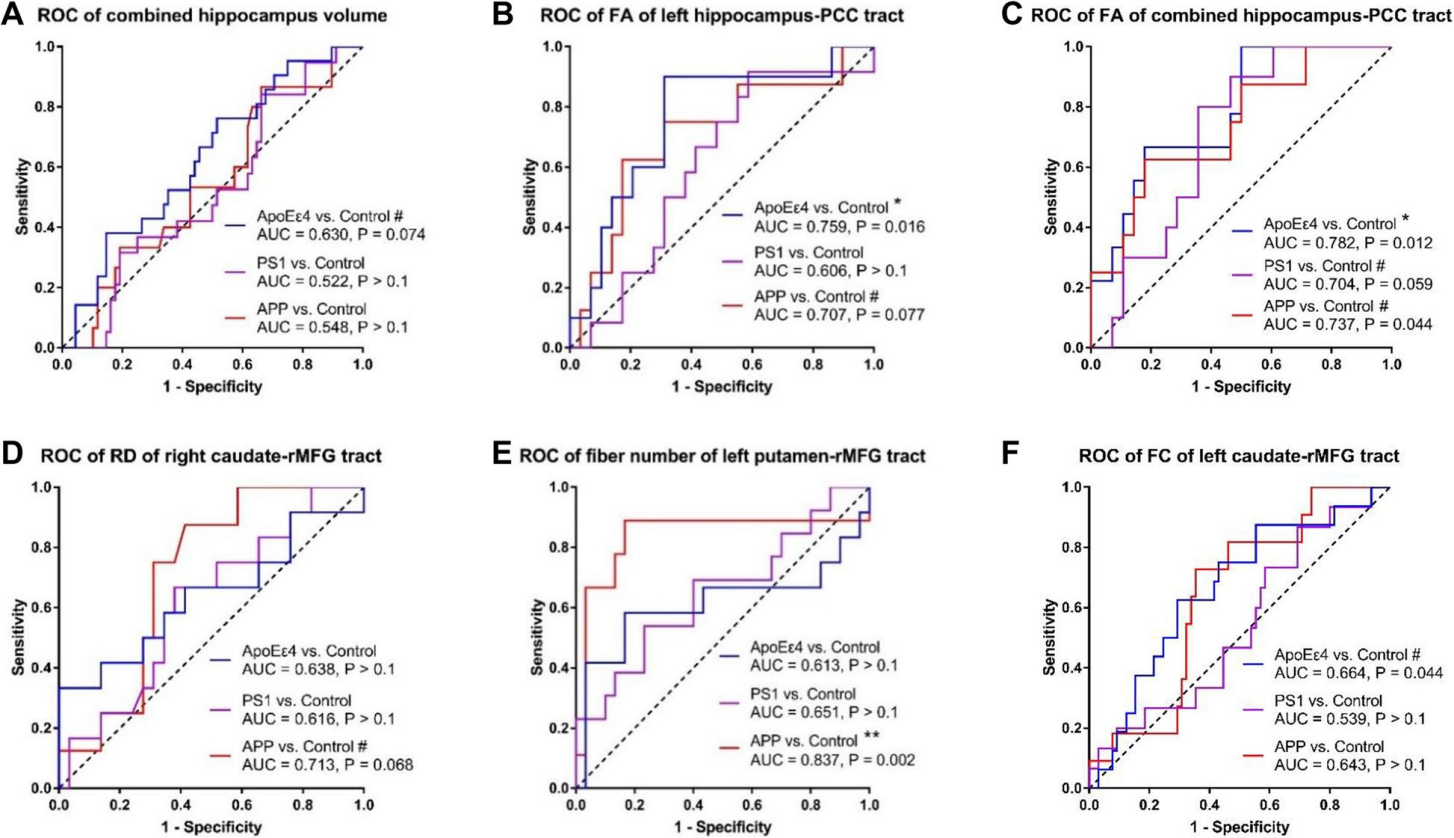
ROC curves of neuroimaging measures that showed group difference. # 0.017 < *P* < 0.1, * 0.01 < *P* < 0.017, ** 0.001 < *P* < 0.01

The ROC curves of the neuroimaging measures that showed group differences were shown in Fig. 4. The combined hippocampus volume was able to discriminate the *APOEε4* group from the control group to a trend level (AUC = 0.630, *P* = 0.074), but not for the *APP* or *PS1* groups (Fig. 4A). The FA of left hippocampus-PCC tract was able to discriminate the *APOEε4* group from the control group (AUC = 0.759, *P* = 0.016) and to a trend level in discriminating the *APP* group from the control group (AUC = 0.707, *P* = 0.077), but not for the *PS1* groups (Fig. 4B). The FA of combined hippocampus-PCC tract was able to discriminate the *APOEε4* group (AUC = 0.782, *P* = 0.012) from the control group and to a trend level in discriminating the *PS1* groups (AUC = 0.704, *P* = 0.059) and the *APP* group (AUC = 0.737, *P* = 0.044) from the control group (Fig. 4C). The RD of right caudate-rMFG tract was able to discriminate the *APP* group from the control group to a trend level (AUC = 0.713, *P* = 0.068), but not for the *APOEε4* or *PS1* groups (Fig. 4D). The fiber number of left putamen-rMFG tract was able to discriminate the *APP* group from the control group (AUC = 0.837, *P* = 0.002), but not for the *APOEε4* or *PS1* groups (Fig. 4E). The FC of left caudate-rMFG tract was to a trend level in discriminating the *APOEε4* group from the control group (AUC = 0.664, *P* = 0.044), but not for the *APP* or *PS1* groups (Fig. 4F).

### Correlations of imaging measures with neuropsychological measures

**Fig. 5:**
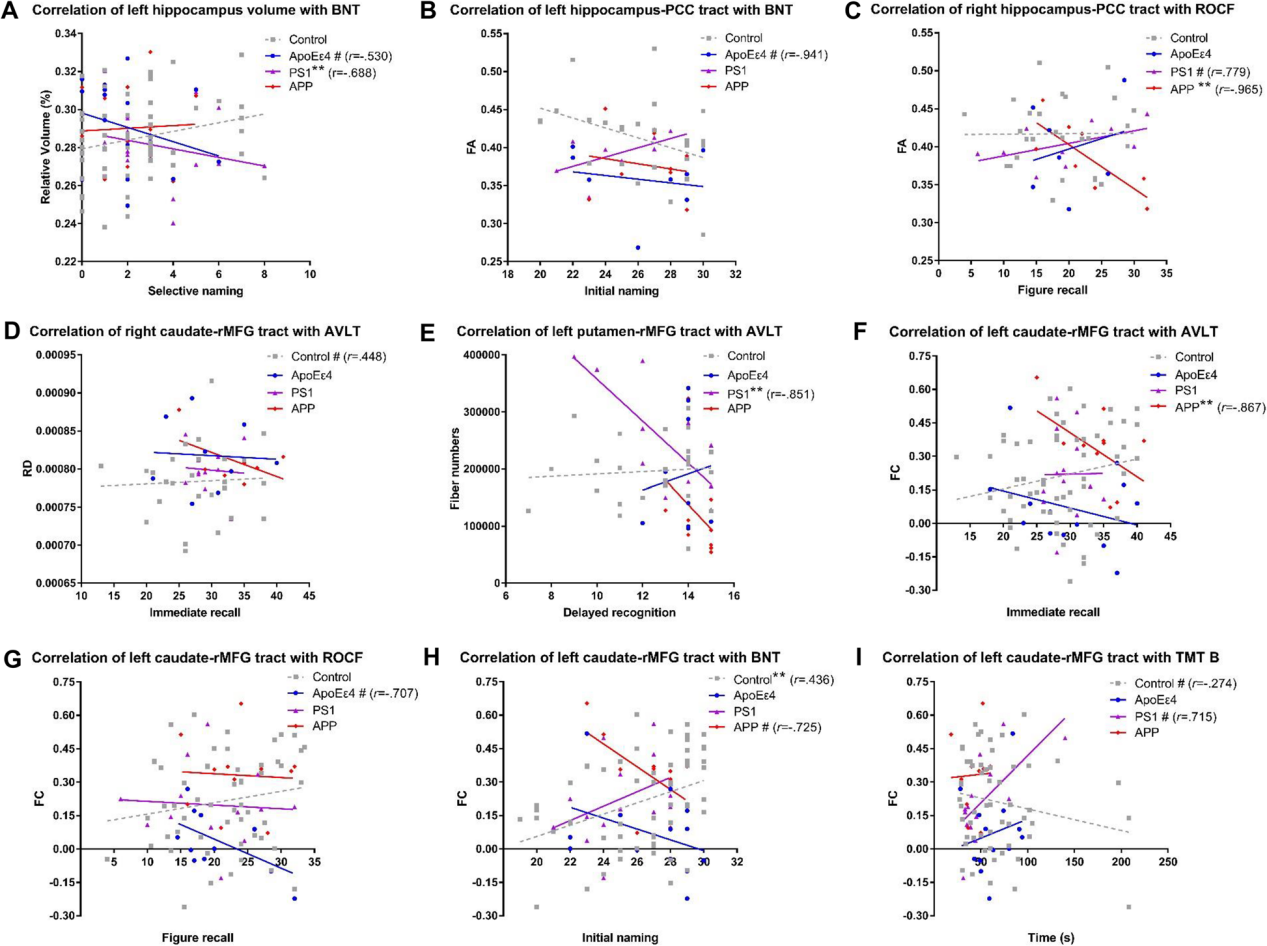
Correlations of neuroimaging measures that showed group difference with neuropsychological measures. Partial correlations controlling for age, sex, and education were performed. The best-linear-fit regression lines are displayed for the convenience of readers. # 0.0125 < *P* < 0.1, * 0.01 < *P* < 0.0125, ** 0.001 < *P* < 0.01

The correlations of imaging measures that showed group differences with specific cognitive function domains were shown in Fig. 5. Relative volume of left hippocampus correlated significantly negatively with BNT selective naming in the *PS1* group (*r* =  − 0.688, *P* = 0.003) and to a trend level in *APOEε4* group (*r* =  − 0.530, *P* = 0.035), but not in the control group (Fig. 5A). The FA of left hippocampus-PCC tracts correlated to a trend level negatively with BNT initial naming in the *APOEε4* group (*r* =  − 0.941, *P* = 0.017), but not in the control group (Fig. 5B). The FA of right hippocampus-PCC tracts correlated significantly negatively with ROCF figure recall in the *APP* group (*r* =  − 0.965, *P* = 0.008) and to a trend level positively in the *PS1* group (*r* = 0.779, *P* = 0.039), but not in the control group (Fig. 5C). RD of right caudate-rMFG tract correlated to a trend level positively with AVLT immediate recall in the control group (*r* = 0.448, *P* = 0.028), but not in any genetic group (Fig. 5D). Fiber number of left putamen-rMFG tract correlated significantly negatively with AVLT delayed recognition in the *PS1* group (*r* =  − 0.851, *P* = 0.004), but not in the control group (Fig. 5E). FC of left caudate-rMFG tract correlated significantly negatively with AVLT immediate recall in the *APP* group (*r* =  − 0.867, *P* = 0.005), but not in the control group (Fig. 5F). Furthermore, it correlated to a trend level negatively with ROCF figure recall in the *APOEε4* group (*r* =  − 0.707, *P* = 0.033), but not in the control group (Fig. 5G). It correlated to a trend level negatively with BNT initial naming in the *APP* group (*r* =  − 0.725, *P* = 0.042), but significantly positively (*r* = 0.436, *P* = 0.001) in the control group (Fig. 5H). In addition, it correlated to a trend level positively with TMT B time in the *PS1* group (*r* = 0.715, *P* = 0.013), but to a trend level negatively (*r* =  − 0.274, *P* = 0.047) in the control group (Fig. 5I).

## Discussion

To the best of our knowledge, this is the first study looking at the common and differentiated effects of *APOEε4* and pathogenic gene mutation on cognitive domains and circuit-based imaging markers in the preclinical stage of FAD. The major findings were that there are neuropsychological and imaging changes in the preclinical stage of FAD. Some are shared by *APOEε4* and known pathogenic gene mutation, which is the decreased white matter integrity of the hippocampus-PCC circuit. Some are unique to *APOEε4* carriers, including increased hippocampal volume and decreased FC of the frontostriatal pathway, which negatively correlated with language, visuospatial, and working memory. These findings paved the way for early identification and development of gene-, domain-, and circuit-targeted prevention strategy.

For neuropsychological measures, our results primarily indicate that verbal episode memory was higher in the *APP* group and was able to distinguish *APP* mutation carriers from non-carriers. Only a few studies have examined neuropsychological measures in the preclinical stage of FAD, and the results were inconclusive. One study found that episode memory and executive functioning were impaired in preclinical *PS1* mutation carriers at 35 years of age, 9 years before estimated age of onset, as compared with non-carriers [6]. We did not find such changes in PS1 mutation carriers, probably due to longer years before estimated onset. Another two studies did not find changes in episode memory at baseline (immediate and 30 min later) but found declined memory retention 7 days later in preclinical mutation carriers as compared with non-carriers [7, 43]. Our results in *PS1* mutation carriers are consistent with them. Some studies found impaired episode memory in *APOEε4* carriers aged 50–59 years [44], while some showed better memory performance in older *APOEε4* carriers without subjective memory impairment [45]. We did not find such changes in *APOEε4* carriers, probably due to a younger age. None of the above studies examined episode memory in preclinical *APP* mutation carriers. Since *APP* group had relatively high education levels, the effect of education on the memory performance could not be ruled out, although it was controlled for during the analysis. Further correlation of recall scores in the AVLT test with education, using age and sex as covariates, showed that delayed recall score correlated significantly positively with education in control group (*r* = 0.293, *p* = 0.018), but not significantly in the *APP* group (*r* = 0.048, *p* = 0.866). In addition, the trend level increased FC of frontostriatal pathway in *APP* group might be an explanation for the biological basis of the increased recall scores in AVLT, since it showed opposite correlations as compared to control group.

For neuroimaging measures, our results indicate that the shared feature of *APOEε4* carriers and *APP*/*PS1* mutation carriers during the preclinical stage is the decreased structural connectivity of the hippocampus-PCC tract. Previous DTI studies showed various patterns of hippocampus-related tracts in the preclinical stage of Alzheimer’s disease. While some studies showed no change of FA or MD of the hippocampus-cingulum tract [8, 37, 46], some showed early decreased MD and later increased MD of the hippocampus [8] in preclinical subjects carrying known pathogenic gene mutation. While some studies showed reduced FA and increased diffusivity of cingulum bundle [47, 48], some showed no change in FA or diffusivity of hippocampus or cingulum bundle [49] in cognitive normal *APOEε4* carriers. Decreased FA and increased MD indicate a reduction in white matter integrity and disruption of white matter microstructures, respectively [46]. We did not find early structural connectivity changes of the frontostriatal pathway in the *APOEε4* group but found reduced fiber numbers and increased RD, especially in the *APP* group, which might reflect an early loss of axons or myelination [37]. Similar findings that the effects in AxD were much smaller than in RD have been reported for healthy *APOEε4* carriers, though not for the same white matter tract [50]. AxD and RD, the principal direction and perpendicular direction of the diffusion ellipsoid, have been shown to assess axonal integrity and myelin integrity, respectively [37]. Altogether, our findings of the commonality of *APOEε4* and *APP*/*PS1* mutation effect on hippocampus-PCC structural connectivity indicate that the disrupted white matter integrity of hippocampus-PCC tract is a promising imaging marker for preclinical Alzheimer’s disease, either familial or sporadic.

In addition to the shared feature, our results indicate the unique features of *APOEε4* carriers are early increased hippocampal volume and decreased FC of frontostriatal pathway. Previous studies have not reached consistence in volume changes of hippocampus in preclinical FAD. While some reported volume reduction [11], some reported no volume change [8, 37]. Regarding hippocampal volume changes in cognitive normal *APOEε4* carriers, some reported decreased volume in healthy older adults [19], some reported no change of the volume in healthy young adults [51] or preclinical Alzheimer’s disease until 50 years [22], and some reported volume increase in healthy older adults without subjective memory impairment [45]. Another study showed that among cognitively normal and early mild cognitive impairment participants, ε4 + status was independently associated with increased cortical thickness especially in limbic regions [52]. Such discrepancy might be due to the differences in subjects’ demographics (such as age and EYO), sample sizes, or image processing methods. Altogether, our finding of trend level increased hippocampal volume might be the unique feature for cognitive normal *APOEε4* carriers with relatively young age in FAD, instead of the pathological process of Alzheimer’s disease.

Regarding the frontostriatal circuit, there is evidence showing different levels of damage to the right DLPFC-right caudate-left thalamus-right DLPFC circuit in different groups of mild cognitive impairment (reversed to normal, stable, or progressed to dementia). Specifically, the connectivity strength of this circuit was damaged in the stable and progressed group, not in the reversed group, indicating that FC of the frontostriatal circuit might be a potential biomarker for early detection of Alzheimer’s disease [53]. Another study found that asymptomatic *APOEε4* carriers showed a slower longitudinal increase in FC in the DLPFC region than non-carriers [54]. Another recent study found lower FC between nucleus accumbens, another striatal subregion, and various cortical regions in cognitive normal elder *APOEε4* carriers, which correlated with increased TNF-α in CSF, implicating neuroinflammation in *APOEε4* carriers [55]. There are also studies finding early compensation in terms of FC in frontal executive regions during aging and at asymptomatic *APP* mutation carriers [37], indicating the enhanced metabolic demand engaged by an adaptive brain for cognitive reserve. Increased connectivity may indicate high processing burden and/or noisy inefficient synaptic communication, as highly connected regions are particularly vulnerable to Aβ deposition because of their increased synaptic activity, according to the “nodal stress” hypothesis [56]. It should be noted that *APOEε4* status can show dose effect on brain functional connectivity in patients with subjective cognitive decline. For example, lower dynamic functional connectivity involving the insular and temporal neocortex was negatively correlated with the number of *APOE* ε4 alleles in patients with subjective cognitive decline [57]. Altogether, our findings of the unique effects of *APOEε4* carriers on this frontostriatal circuit indicated a possible lower capacity for adaptation and higher chance of disease progression, with the potential mechanism of neuroinflammation rather than inefficient synaptic communication.

For the correlations of imaging with neuropsychological measures, our findings suggest that the hippocampus-related imaging markers are differentially associated with language and visual episode memory in *APOEε4* carriers and *APP/PS1* mutation carriers. One study found reduced hippocampus volume accompanied by better performance in BNT in cognitive normal *APOEε4* carriers [19], indicating the negative association of hippocampus volume with language. Our result in *APOEε4* carriers is consistent with it, though the changes were in the opposite direction probably due to young age. Other studies found that hippocampus and PCC regions are involved in visual episodic memory in asymptomatic *PS1* mutation carriers [58] and *APOEε4* carriers [59]. Higher activation of the hippocampus, less deactivation of PCC, and reduced connectivity of the hippocampus and cingulum are associated with worse memory performance. Our results showed the opposite association of hippocampus-PCC structural connectivity with visual episode memory in *PS1* and *APP* mutation carriers probably indicating a different mechanism. Our results further indicated that verbal episode memory that primarily improved in *APP* mutation carriers requires adaptive changes of the function while impairing structural connectivity of the frontostriatal pathway. The FC of the caudate-rMFG tract plays important role in various cognitive domains, including episode memory, language, and executive function in healthy controls, and such associations were disrupted in *APOEε4* and *APP/PS1* mutation carriers. Previous studies also support that frontostriatal regions are involved in executive function, language, working memory, and memory binding in FAD [15, 58]. Our findings deepened current knowledge and indicated the association of FC of the caudate-rMFG tract with different cognitive domains, which might serve as a promising marker for early identification of preclinical Alzheimer’s disease.

This study has several limitations. First, the sample sizes are relatively small, especially for DTI data, and the low numbers of *APOE* ε4/ε4 and ε4/ε2 subjects make it impossible to explore the gene dose effect. Second, the correlation analyses did not correct for the number of cognitive domains; thus, the trend level findings might be false positive and need to explain with caution. Third, we selected regions and tracts of interest, instead of looking at circuits or networks in the whole brain. Fourth, the neuropsychological batteries and imaging techniques are still developing; thus, the accuracy needs to be validated and compared with other advanced methods. Future studies will enlarge the sample size and use a longitudinal design to evaluate the gene-specific effect on the trajectory of neuropsychological and imaging markers as well as cross-validate in the sporadic population at risk for Alzheimer’s disease.

## Conclusions

There are neuropsychological and imaging changes in the preclinical stage of FAD. The unique imaging markers in *APOEε4* carriers are early elevation of hippocampal volume and decreased FC of the caudate-rMFG tract. The shared imaging marker for *APOEε4* and *APP/PS1* mutation carriers is disrupted structural integrity of the hippocampus-PCC pathway, which plays important role in language and visual episode memory. FC of the caudate-rMFG tract plays important role in various cognitive domains including verbal episode memory, language, and executive function. These findings are helpful for the early identification of Alzheimer’s disease and the development of generalized and individualized prevention and intervention strategy.

## Supplementary Information

**Additional file 1:** **Table S1.** Subject Demographic and Clinical Data of APOEε4 subgroups.

## Data Availability

Both raw and processed data that support the findings of the current study will be made available upon request to the corresponding author and the CFAN and COAST committee to ensure that the privacy of the participants is protected.

## Abbreviations

*APOE*: Apolipoprotein E gene
*APP*: Amyloid precursor protein
AUC: Area under the curve
AVLT: Auditory Verbal Learning Test
AxD: Axial diffusivity
BNT: Boston naming test
CDR: Clinical Dementia Rating scale
DTI: Diffusion tensor imaging
EYO: Estimated years from symptom onset
FA: Fractional anisotropy
FC: Functional connectivity
MD: Mean diffusivity
MMSE: Mini-Mental State Examination
MoCA: Montreal Cognitive Assessment
PCC: Posterior cingulate cortex
*PS1*: Presenilin 1
*PS2*: Presenilin 2
RD: Radial diffusivity
rMFG: Rostral middle frontal gyrus
ROCF: Rey-Osterrieth complex figure test
ROI: Region of interest
rsfMRI: Resting state functional MRI
TMT: Trail making test
