# Supplementary material for: Shared and unique effects of ApoEε4 and pathogenic gene mutation on cognition and imaging in preclinical familial Alzheimer’s disease
Source: Alzheimers Res Ther. 2023 Feb 28;15:40. doi: 10.1186/s13195-023-01192-y (PMC9972804; doi:10.1186/s13195-023-01192-y)
Supplement: Supplementary file 1 — Additional file 1: Table S1. Subject Demographic and Clinical Data of APOEε4 subgroups. [file 13195_2023_1192_MOESM1_ESM.docx]

***Additional file 1***

**Shared and unique effects of *ApoEε4* and pathogenic gene mutation on cognition and imaging in preclinical familial Alzheimer’s disease**

Meina Quan^1-6^, Qi Wang^1-6^, Wei Qin^1-6^, Wei Wang^1-6^, Fangyu Li^1-6^, Tan Zhao^1,2^, Tingting Li^1-6^, Qiongqiong Qiu^1-6^, Shuman Cao^1-6^, Shiyuan Wang^1-6^, Yan Wang^1-6^, Hongmei Jin^1-6^, Aihong Zhou^1-6^, Jiliang Fang^7^, Longfei Jia^1-^,^6^ Jianping Jia^1-6^.

^1^ Innovation Center for Neurological Disorders and Department of Neurology, Xuanwu Hospital, Capital Medical University, Beijing, China;

^2^ National Center for Neurological Disorders and National Clinical Research Center for Geriatric Diseases, Beijing, China;

^3^ Clinical Center for Neurodegenerative Disease and Memory Impairment, Capital Medical University, Beijing, China;

^4^ Beijing Key Laboratory of Geriatric Cognitive Disorders, Beijing, China;

^5^ Center of Alzheimer's Disease, Beijing Institute for Brain Disorders, Beijing, China;

^6^ Key Laboratory of Neurodegenerative Diseases, Ministry of Education, Beijing, China

^7^ Guang’anmen Hospital, China Academy of Chinese Medical Sciences, Beijing, China.

**Table S1.** Subject Demographic and Clinical Data of *APOEε4* subgroups

|  | ***APOE*ε4/ε4**  **(n=1)** | ***APOE*ε4/ε3**  **(n=22)** | ***APOE*ε4/ε2**  **(n=3)** | ***P*-value** |
| --- | --- | --- | --- | --- |
| Age (years) | 44.00 (0.00) | 43.45 (12.58) | 52.67 (16.17) | >0.05 |
| Sex (male/female) | 1/0 | 11/11 | 0/3 | >0.05 |
| Education (years) | 14.00 (0.00) | 11.32 (4.95) | 14.33 (1.16) | >0.05 |
| EYO (years) | -24.00 (0.00) | -15.05 (9.56) | -20.67 (12.58) | >0.05 |
| MMSE | 29.00 (0.00) | 28.95 (1.64) | 30.00 (0.00) | >0.05 |
| MoCA | 25.00 (0.00) | 27.25 (2.69) | 28.33 (0.58) | >0.05 |

For continuous variables, data are shown in mean (SD), and one-way ANOVA with post-hoc between-group comparisons using Bonferroni analysis; for categorical variables, chi-square test was used to compare between groups. *0.01<*P*<0.05, **0.001<*P*<0.01.
